# Predictors and reference equations for augmentation index, an arterial stiffness marker, in healthy children and adolescents

**DOI:** 10.6061/clinics/2021/e2350

**Published:** 2021-01-11

**Authors:** Luzia Maria dos Santos, Isabel Cristina Gomes, José Felippe Pinho, Claudia Marotta Neves-Alves, Giselle Santos Magalhães, Maria José Campagnole-Santos, Maria da Glória Rodrigues-Machado

**Affiliations:** I Programa de Pos-Graduacao em Ciencias da Saude, Faculdade Ciencias Medicas de Minas Gerais, Belo Horizonte, MG, BR; II Departamento de Fisiologia e Biofisica, Instituto de Ciencias Biologicas, Universidade Federal de Minas Gerais, Belo Horizonte, MG, BR

**Keywords:** Pulse Wave Velocity, Pulse Pressure, Hemodynamic Parameters, Quality of Life, Arterial Stiffness, Children, Adolescents, Anthropometry

## Abstract

**OBJECTIVES:**

To investigate predictors and propose reference equations for the augmentation index normalized to 75 bpm heart rate (AIx@75) in healthy children and adolescents.

**METHODS:**

This was a cross-sectional, observational study involving 134 healthy children and adolescents aged 9 to 19 years old. Participants were categorized into child (n=53) and adolescent (n=81) groups, as well as into male (n=69) and female (n=65) groups. We evaluated AIx@75, vascular and hemodynamic parameters, anthropometric data, physical activity profile, and quality of life (Peds-QL4.0; physical, emotional, social and school domains).

**RESULTS:**

The predictors of AIx@75 in the whole sample were age, peripheral diastolic blood pressure (pDBP), mean arterial pressure, pulse pressure amplification (PPA), systolic volume (SV), cardiac index (CI), and pulse wave velocity (PWV; R2=80.47%). In the male group, the predictors of AIx@75 were SV, CI, total vascular resistence (TVR), and PWV (R2=78.56%), while in the female group, they were pDBP, PPA, SV, and PWV (R2=82.45%). In the children, they were pDBP, PPA, SV, and PWV (R2=79.17%), while in the adolescents, they were body mass index, pDBP, PPA, SV, TVR, and PWV (R2=81.57%).

**CONCLUSION:**

In the present study, we used a representative sample from Belo Horizonte to establish normality values of AIx@75. We also identified, for the first time, independent predictors of AIx@75 in healthy children and adolescents categorized by sex and age. Determining AIx@75 reference equations may facilitate the early diagnosis of preclinical atherosclerosis and allow an objective measure of the vascular effects of therapeutic interventions aimed at modifying cardiovascular risk factors.

## INTRODUCTION

Evidence indicates that atherosclerosis begins in childhood and that risk factors for cardiovascular disease, including diabetes mellitus, hypertension, dyslipidemia, and obesity, are increasing among children and adolescents (1). The main markers of arterial structural change are compliance, distensibility, and stiffness. Arterial stiffness can be assessed using central systolic blood pressure (cSBP), augmentation index (AIx), and pulse wave velocity (PWV) (2,3). The AIx is calculated from the central arterial pulse wave, which comprises a forward wave generated by left ventricular ejection and a backward wave that depends on the elastic properties of the arterial tree, the wave transmission velocity, and the distance from the reflection sites. Aortic augmentation pressure (aAP) refers to the absolute increase in aortic systolic blood pressure resulting from the reflection wave. It is an important physiological phenomenon as it maintains arterial blood flow and increases the blood supply to the coronary arteries (4). The AIx depends on the relationship between aAP and central PP. AIx elevation results in increased left ventricular afterload and oxygen consumption in the myocardium, as well as decreased myocardial perfusion pressure, inducing an imbalance in the supply-demand relationship, which is especially dangerous in hypertrophied hearts with coronary artery disease (5).

AIx is an independent predictor of future cardiovascular events and all-cause mortality (6). It has been evaluated in healthy children and adolescents (7-9) and is increased in various diseases, such as arterial hypertension (10), chronic kidney disease (11), diabetes (12), asthma (13) and obesity (14-16).

In different populations, AIx reference equations have been proposed to assess arterial stiffness in children and adolescents (8,17). Because the AIx is influenced by age, sex, height, and heart rate (18), as well as by ethnicity (19), researchers must ascertain the predictive factors and establish reference equations for the AIx in Brazilian children and adolescents. Such equations may facilitate the early diagnosis of subclinical atherosclerosis and allow an objective measure of the vascular effects of therapeutic or non-drug interventions aimed at modifying cardiovascular risk factors.

## MATERIALS AND METHODS

This was a cross-sectional study evaluating the AIx corrected for a heart rate of 75 bpm (AIx@75) in healthy children and adolescents aged 9 to 19 years who had a normal body mass index (BMI). Initially, 170 students were recruited from schools of nine administrative regions in Belo Horizonte, MG, between June 2016 and March 2017. Thirty-six of these were then excluded: seven did not attend on the day scheduled for the examination, nine did not complete the informed consent form, 15 had a history of chronic respiratory disease (recurrent wheezing or asthma), three used medications for epilepsy, and two used Ritalin. The final sample consisted of 65 female and 69 male participants.

### Sample calculation

**Figure 1:**
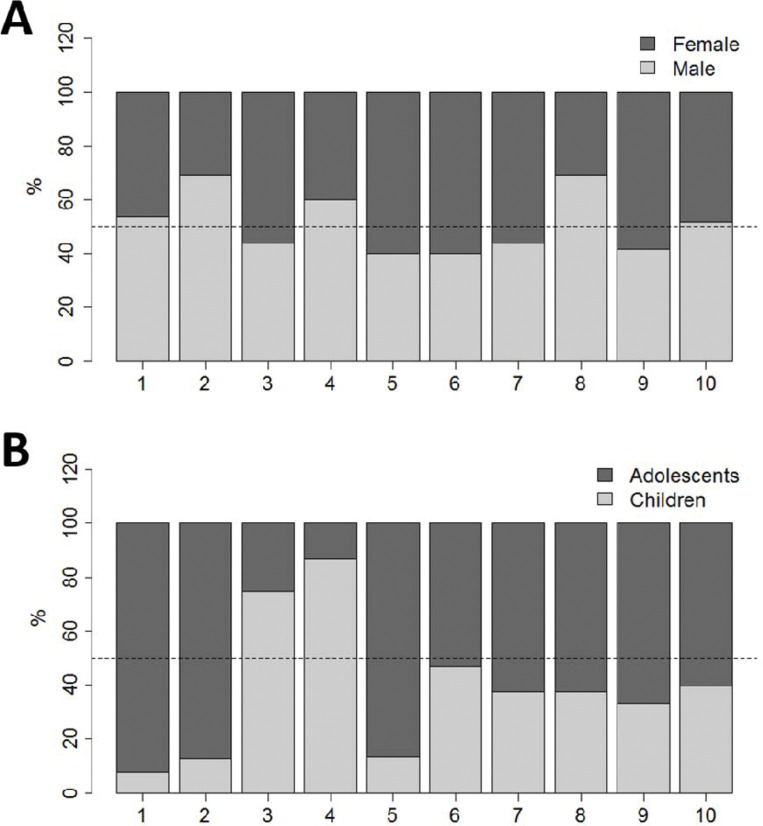
Distribution of sampled individuals in nine administrative regions of Belo Horizonte by (a) sex and (b) age group (children, 9-12 years and adolescents, 13-19 years).

We calculated the necessary sample size to test the correlation of an anthropometric variable (weight) with arterial stiffness in adolescents. Based on the correlation in an earlier study (20), at 5% significance and 90% power, at least 121 individuals were required (21), and the sample was planned to ensure it was representative of all city regions, sexes, and age groups. Figure 1 shows the distribution of the sample according to city region, sex, and age group.

The present study was approved by the Ethics and Research Committee of FCM-MG (CAE: 48326715.5.0000.5134) and by the Minas Gerais State Department of Education. Parents or guardians provided written informed consent.

### Anthropometric evaluation

Children and adolescents with a BMI percentile between 5% and 85% were included in the study. Waist and hip circumferences (WCs and HCs) were evaluated, and waist-to-hip ratio and waist-to-stature ratio were calculated. The World Health Organization (WHO) considers waist-to-hip ratio as one criteria for characterizing metabolic syndrome and cardiovascular risk, with cutoff values of 0.90 for men and 0.85 for women (WHO, 2006) (22).

### Assessment of baseline physical activity level

Baseline physical activity was assessed using the Physical Activity Questionnaire-Child (IPAQ-C) short version, which measures the level of physical activity of children and adolescents in the week prior to the questionnaire (23).

### Quality of life assessment

Health-related quality of life was evaluated and analyzed using the Pediatric Quality of Life Inventory version 4.0 (Peds-QL4.0), which comprises 23 questions that assess the perception of children and adolescents in the following dimensions in the last month: physical (eight items); emotional - cognitive and intellectual (five items); social (five items), and school (five items) (24). In the present study, the children and adolescents were stratified according to this questionnaire.

### Assessment of respiratory health

The respiratory health of the participants was evaluated using the International Study of Asthma and Allergies in Childhood (ISAAC) questionnaire. We excluded volunteers with a significant respiratory history or medical diagnosis of asthma, which is a prevalent disease in this age group (25).

### Socioeconomic classification

Socioeconomic class was determined as recommended by the Brazilian Association of Research Companies (ABEP, 2015) (26).

### Assessing arterial stiffness and vascular/hemodynamic parameters

**Figure 2:**
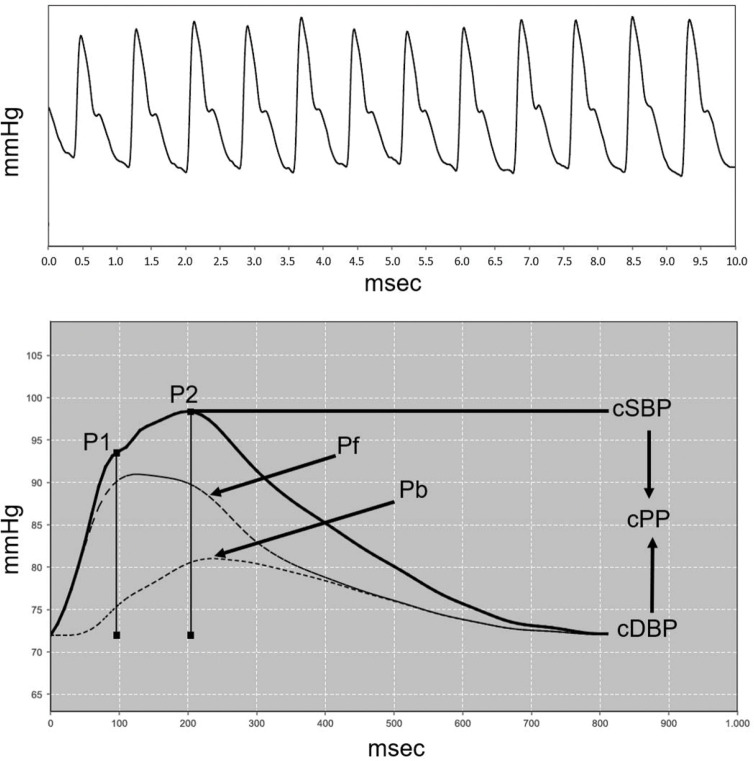
Pulse waves from the brachial (A) and aortic (B) arteries in a female teenager. P1=First systolic peak; P2=Second systolic peak; Pf=Forward or ejection wave; Pb=Backward or reflection wave; cSBP and cDBP=central systolic and diastolic blood pressure; cPP=central pulse pressure. AIx@75=(P2-P1)/cPP*100 corrected for a heart rate of 75 bpm.

The Mobil-O-Graph^®^ - Pulse Wave Analysis Monitor (IEM, Stolberg, Germany) was used to assess the arterial stiffness indexes, as well as the vascular and hemodynamic parameters (11,12). The PWV was determined using a mathematical model that considered several parameters to analyze pulse wave and wave separation. The AIx@75 was calculated as the pressure difference between the second peak of the reflection wave (P2) and the first peak of the incident wave (P1). It was expressed as a percentage of central pulse pressure (PPc) (AIx@75 = [P2 - P1]/PPc x 100]) (16). In addition to the arterial stiffness indexes, the following peripheral vascular parameters were evaluated: systolic and diastolic blood pressure (pSBP and pDBP), pulse pressure (pPP), central arterial blood pressure (cSBP, cDBP, cPP), heart rate (HR) and hemodynamic parameters (cardiac output [CO]), total vascular resistance (TVR), and cardiac index (CI). Figure 2 shows pulse waves from the brachial and aortic arteries in a female teenager.

Both pSBP and pDBP were classified according to the recommended percentile of each age group: a blood pressure less than the 90^th^ percentile is normal; between the 90^th^ and 95^th^ percentile denotes prehypertension. In adolescents, blood pressure equal to or exceeding 120/80 mmHg constitutes prehypertension, even if this figure is less than the 90^th^ percentile, according to the VI Brazilian Guidelines for Hypertension and the recommendations of the National Institutes of Health National Heart. Therefore, a new categorization of normal and elevated blood pressure was defined by age, height, and sex according to the percentile (27,28).

### Statistical analysis

Categorical variables are presented as counts and percentages, while continuous variables are given as mean ± standard deviation. Continuous variables were evaluated using the Shapiro-Wilk normality test. The Student’s t-test and Mann-Whitney test were used to compare the means of independent samples. To evaluate the association between categorical variables, the chi-square test of independence was used. Association between numerical variables was evaluated using the Pearson and Spearman linear correlation coefficients.

Four observations were excluded because they were AIx@75 outliers (>3 standard deviations from the mean). To derive AIx@75 prediction equations, linear regression models were constructed for the whole sample according to the sex and age groups. Variables that had *a p*-value <0.20 in the analysis of association with AIx@75 were included in the saturated model. The stepwise strategy was then applied to reach the final model. Quality of fit was assessed using the adjusted R^2^, by residue analysis (evaluation of normality, homoscedasticity, independence, and influential), and by evaluating multicollinearity. To validate the equations internally, 5,000 bootstrap samples were constructed; the average squares of the residuals were calculated and compared to those obtained in the proposed models. All analyses were carried out in the free program R version 3.3.2, and a significance level of 5% was adopted.

## RESULTS

Figure 1A presents the percentage distribution of the female and male groups in the nine administrative regions of Belo Horizonte, while Figure 1B represents the percentage distribution of the children (9 to 12 years) and adolescents (13 to 19 years) in the same regions.

The female and male groups were similar in all evaluated parameters, while the HC was significantly higher in the adolescent group (Table 1).

The pPP was significantly higher in the female group than in the male group, while the TVR significantly lower (Table 2).

Table 3 shows how AIx@75 was associated with anthropometric characteristics, physical activity profile, and quality of life in the whole sample, taking into account sex and age range. The AIx@75 did not correlate with sex, socioeconomic class, or physical activity profile in either children or adolescents. In the whole sample, the AIx@75 correlated negatively with weight, height, HC, and physical aspect. Meanwhile, it correlated positively with WC/stature in the whole sample and in the male group. In the male group, the AIx@75 correlated negatively with height and physical aspect. In the female group, the AIx@75 correlated negatively with weight, height, and BMI. In the children, the AIx@75 correlated negatively with weight, height, BMI, and physical aspect. The AIx@75 of the adolescents negatively correlated with weight, height, and HC.

Table 4 shows the linear correlation coefficients of the AIx@75 with the peripheral and central arterial blood pressure, hemodynamic parameters, and arterial stiffness.

The adjusted regression models are presented in Table 5. For all models, the residues met the assumptions of normal distribution, independence, and homoscedasticity. None of the models presented multicollinearity problems. In the internal validation, absolute optimism of 0.068-0.129 was obtained for the constructed models. The predictors of AIx@75 in the whole sample group were age, pDBP, peripheral mean arterial pressure (pMAP), pulse pressure amplification (PPA), SV, CI, and PWV, (R2=80.47%). In the male group, the predictors of AIx@75 were pPP, SV, CI, TVR, and PWV (R2=78.56%), while in the female group, they were pDBP, PPA, SV, and PWV (R2=82.45%). In the children, they were pDBP, PPA, SV, and PWV (R2=79.17%), while in the adolescents, they were BMI, pDBP, PPA, SV, TVR, and PWV (R2=81.57%).

## DISCUSSION

The present study was the first to evaluate the independent predictors of the AIx@75 and to propose reference equations in a representative population from the city of Belo Horizonte. Several authors have suggested that age-related changes in the AIx@75 are more evident in younger subjects. Conversely, the increase in PWV is more significant in individuals aged more than 50 years old (8,4).

In the present study, PWV did not differ between the groups, ranging from 4.5 to 4.59 ms, similar to the values found by Hivédgi et al. (8) in healthy children and adolescents aged 3-18 years. However, PWV was related to the AIx@75 in all groups in the present study, corroborating previous literature showing that the AIx@75 partly depends on aortic and large-artery PWV. Specifically, higher PWV values lead to early arrival of reflected waves (29).

Some evidence has demonstrated that the measured cSBP, which is the pressure exerted on vital organs such as the brain, heart, and kidneys, differs from the measured brachial pressure because of pressure wave amplification. The cSBP is considered a better predictor of cardiovascular events and target organ injury than peripheral measurement (4,30,31). In the present study, no differences were observed between the various components of central blood pressure in the groups studied, and the peripheral variables pSBP, pDBP, and pMAP did not differ between the groups, while pPP was significantly higher in the female group. Although, pSBP correlated negatively with the AIx@75 in all groups, pSBP did not contribute to the stepwise multiple regression equation of any group.

In the present study the PPA (pPP/cPP ratio) was similar between the groups. This index denotes an important physiological function protecting the heart against afterload and guarding the microcirculation from pulsatile pressure stress. Reductions in PPA are associated with cardiovascular risk factors such as aging and hypertension. Moreover, PPA is inversely related to arterial stiffness, organ damage, and mortality (32). In the present study, PPA influenced the AIx@75 in all groups except the male sex group.

The hemodynamic variables of SV and CI did not differ between the groups. SV was negatively correlated and influenced AIx@75 in all groups, while TVR was significantly lower in the female than in the male group. As expected, the AIx@75 was correlated positively with TVR in all groups, but influenced only the male and adolescent groups. SV was negatively correlated with the AIx@75 and contributed to the stepwise multiple regression equation of the AIx@75 in all groups.

Several studies have shown that the AIx@75 increases with age in normotensive individuals (8,4). In the present study, the population was categorized by age as children (9-12 years) and adolescents (13-19 years), and the AIx@75 did not differ between these two groups. This is in contrast to the results of Hivédgi et al. (8), probably because those authors included healthy children and adolescents aged 3-18 years, while the present study only included children above 9 years of age (8). In the final stepwise multiple regression model, age only influenced the AIx@75 in the whole sample group.

One determining factor of AIx@75 is height (8). Shorter individuals have a lower arterial tree and wave reflection sites closer to the heart. The earlier arrival of the reflection wave favors an increase in cSBP and therefore in AIx@75. In the present study, the AIx@75 and height did not differ between the groups, but a negative correlation was observed between the AIx@75 and height of participants in all groups studied. However, in the final stepwise multiple regression model, height did not influence the AIx@75.

Although anthropometric measures of body composition (WC, HC, WC/HC and WC/stature) are used as indicators of cardiovascular risk (33), these variables did not influence the AIx@75 in any of the groups analyzed in the present study, suggesting that, in healthy children and adolescents with normal BMI, anthropometric measures of body composition are not good predictors of arterial stiffness.

Obesity in children and adults is associated with a high prevalence of cardiovascular diseases. In the present study, the weight of the participants was similar in the four groups evaluated, and it was within the z score for age. However, the AIx@75 correlated negatively with the weight of the participants among the whole sample, as well as in the female, child, and adolescent groups. In the multiple regression model, weight did not influence the AIx@75. Similarly to weight and stature, BMI did not differ between the groups studied, while the BMI of the female and child groups correlated negatively with the AIx@75. In the stepwise multiple regression model, BMI only influenced the AIx@75 in the adolescent group. Further studies with more accurate measures of muscle mass and fat percentage may provide more information regarding the AIx@75, since body fat distribution may affect arterial compliance and glucose metabolism.

Diseases have a negative impact on the quality of life of children and adolescents, and evaluation instruments should encompass the physical, psychological (cognitive and emotional), and social dimensions of the patient, as outlined by the World Health Organization. No studies have yet focused on correlating quality of life with arterial stiffness in healthy children and adolescents. In the present study, quality of life was assessed using the Peds-QL 4.0, which evaluates the physical, emotional, social, and school domains. The scoring domains evaluated were similar in all groups. However, the AIx@75 was inversely correlated with the physical domain in the whole sample, the male group, and the children. This domain evaluates the perception of health, correlating it with the capacity to perform daily activities that demand physical effort in the last month. In this present study, none of the domains influenced the AIx@75.

Our study had several strengths. The sample was representative of the different regions of the Belo Horizonte city, sex, and age groups. The adjusted models considered the assumptions necessary to construct linear regression models (normal distribution, independence, and homoscedasticity of residues) and presented no multicollinearity problem. However, the study also had some limitations. Although the sample was adequate, it may have been too small for subgroup analysis. Another limitation was the lack of family history data for cardiovascular disease and dietary habits.

In the present study, we established normality values and identified, for the first time, the independent predictors of the AIx@75 in healthy children and adolescents in a representative sample from Belo Horizonte, categorized by sex and age. Determining the AIx@75 reference equations may facilitate the early diagnosis of subclinical atherosclerosis and allow an objective measure of the vascular effects of therapeutic interventions aimed at modifying cardiovascular risk factors.

## AUTHOR CONTRIBUTIONS

Santos LM collected all data, carried out the initial analysis, interpreted the data, drafted the initial manuscript, and reviewed the manuscript. Gomes IC conceived and designed the study, carried out the analysis, and reviewed the manuscript. Pinho JF, Neves-Alves CM and Magalhães GS contributed to the collection of data, drafted the initial manuscript, and reviewed themanuscript. Campagnole-Santos MJ drafted the initial manuscript and critically reviewed the manuscript for important intellectual content. Rodrigues-Machado MG conceived and designed the study, coordinated and supervised data collection, analyzed and interpreted the data, drafted, edited and critically reviewed the manuscript for important intellectual content. All authors approved the final manuscript as submitted and agree to be accountable for all aspects of the research.

## Figures and Tables

**Table 1:** Anthropometric characteristics, social class, physical activity profile, and quality of life of the individuals in the sample.

		*Sex group*			*Age group*		
Variables	Sample (n=134)	Male (n=69)	Female (n=65)	*p*-value	Child (n=53)	Adolescent (n=81)	*p-*value
Sex, M (%)	69 (51.5%)	69 (100%)	-	-	28 (52.8%)	41 (50.6%)	NS^Q^
Age (years)	13.53±3.17	13.72±3.30	13.32±3.03	NS^W^	10.09±1.02	15.78±1.75	<0.001^W^
Weight (kg)	47.23±12.47	47.79±12.77	46.63±12.22	NS^T^	45.22±11.88	48.54±12.75	<0.20^W^
Height (cm)	156.01±14.73	156.32±14.90	155.68±14.66	NS^T^	153.80±15.27	157.45±14.27	<0.20^T^
BMI (kg/m^2^)	18.96±2.43	19.04±2.51	18.88±2.36	NS^W^	18.78±2.01	19.08±2.67	NS^W^
WC (cm)	71.85±10.47	73.27±9.90	70.34±10.92	NS^W^	72.11±12.54	71.67±8.94	NS^W^
HC (cm)	78.87±12.95	78.45±12.95	79.32±13.03	NS^T^	75.59±12.13	81.02±13.09	0.016^T^
WC/HC	0.87±0.18	0.89±0.20	0.85±0.16	NS^W^	0.89±0.19	0.86±0.17	NS^W^
WC/stature	0.45±0.07	0.45±0.06	0.44±0.07	NS^W^	0.45±0.08	0.44±0.06	NS^W^
Socioeconomic class				NS^Q^			NS^Q^
A or B	60 (44.8%)	30 (43.5%)	30 (46.2%)		24 (45.3%)	36 (44.4%)	
C	74 (55.2%)	39 (56.5%)	35 (53.8%)		29 (54.7%)	45 (55.6%)	
Physical activity profile				<0.20^Q^			NS^Q^
Active	94 (70.1%)	44 (63.8%)	50 (76.9%)		35 (66%)	59 (72.8%)	
Sedentary	40 (29.9%)	25 (36.2%)	15 (23.1%)		18 (34%)	22 (27.2%)	
Quality of life							
Physical score	84.53±13.31	83.39±14.53	85.74±11.87	NS^W^	84.07±14.48	84.84±12.56	NS^W^
Emotional score	71.92±20.50	71.07±21.62	72.83±19.38	NS^W^	74.28±22.03	70.38±19.43	<0.20^W^
Social score	88.45±11.66	88.99±10.17	87.87±13.13	NS^W^	87.89±11.15	88.82±12.04	NS^W^
Educational score	79.37±15.79	79.93±16.44	78.77±15.17	NS^W^	82.15±15.69	77.55±15.69	<0.20^W^

Data are expressed as mean ± standard deviation.

BMI - body mass index; WC - waist circumference; HC - hip circumference; NS - not significant (*p*>0.20).

WC/HC is the ratio between waist and hip circumferences.

WC/stature is the ratio between waist circumference and stature.

A, B and C refer to socioeconomic categories Brazilian Association of Research Companies (ABEP, 2015).

The *p*-values refer to the following tests:

Q qui-square of independence.

W Mann-Whitney and

T Student's t-test for independent sample.

**Table 2:** Parameters of peripheral and central arterial pressure. hemodynamic parameters, and arterial stiffness.

		*Sex groups*			*Age groups*		
Variables	Sample (n=134)	Male (n=69)	Female (n=65)	*p-*value	Child (n=53)	Adolescent (n=81)	*p-*value
Peripheral arterial pressure							
pSBP (mmHg)	111.58±8.18	110.90±7.83	112.29±8.53	NS^T^	110.27±8.69	112.43±7.75	<0.20^T^
pDBP (mmHg)	69.59±6.17	70.10±6.71	69.06±5.53	NS^T^	69.64±6.13	69.56±6.23	NS^T^
pMAP (mmHg)	88.21±8.08	87.89±9.59	88.55±6.14	NS^W^	86.78±10.10	89.15±6.32	<0.20^W^
pPP (mmHg)	42.22±7.42	40.85±7.45	43.68±7.15	0.039^W^	41.13±8.93	42.93±6.19	<0.20^W^
HR (bpm)	79.57±11.51	79.72±12.09	79.41±10.94	NS^T^	79.97±11.58	79.30±11.52	NS^T^
Central blood pressure							
cSBP (mmHg)	98.81±6.90	98.85±7.22	98.76±6.59	NS^T^	98.92±6.02	98.73±7.45	NS^T^
cDBP (mmHg)	71.03±6.19	71.42±6.87	70.62±5.40	NS^T^	70.98±5.65	71.06±6.56	NS^T^
cPP (mmHg)	28.05±5.06	27.53±5.13	28.62±4.97	NS^T^	27.92±5.54	28.14±4.76	NS^T^
PPA	1.53±0.17	1.51±0.15	1.55±0.19	NS^W^	1.50±0.15	1.55±0.19	<0.20^W^
Hemodynamics							
SV (mL)	57.01±11.44	56.56±11.37	57.49±11.57	NS^W^	56.57±12.11	57.31±11.04	NS^W^
CO (l/min)	4.46±0.51	4.39±0.49	4.52±0.53	<0.20^W^	4.40±0.58	4.49±0.46	<0.20^W^
TVR (s*mmHg/mL)	1.22±0.12	1.24±0.12	1.20±0.10	0.007^W^	1.23±0.11	1.21±0.12	NS^W^
CI (L/min*L/m^2^)	3.22±0.58	3.19±0.65	3.25±0.50	NS^W^	3.22±0.61	3.22±0.56	NS^W^
Arterial stiffness							
AP (mmHg)	5.85±2.64	6±2.70	5.69±2.58	NS^W^	5.51±2.33	6.07±2.81	NS^W^
AIx@75	22.21±7.96	22.60±8	21.80±7.97	NS^T^	21.48±8.27	22.70±7.76	NS^T^
PWV (m/s)	4.54±0.27	4.50±0.25	4.59±0.28	<0.20^W^	4.50±0.26	4.57±0.28	NS^W^

Data expressed as mean ± standard deviation. SBP - Systolic blood pressure; DBP - Diastolic blood pressure; MAP - Mean arterial pressure; PP - Pulse pressure; HR - Heart rate; PPA - Pulse pressure amplification (PPp/PPc ratio); AP - augmentation pressure; SV - Systolic volume; CO - Cardiac output; TVR - Total vascular resistance; IC - Cardiac index; AIx@75 - Augmentation index normalized to heart rate of 75 bpm; PWV - Pulse wave velocity; NS - Not significant (p>0.20). The *p*-values refer to the following tests:

W Mann-Whitney and

T Student's t-test for independent samples.

**Table 3:** Evaluating how AIx@75 is associated with anthropometric characteristics. physical activity profile, and quality of life.

		*Sex groups*		*Age groups*	
Variables	Sample (n=134)	Male (n=69)	Female (n=65)	Child (n=53)	Adolescent (n=81)
Sex⊄					
Male	22.59±7.99^T^	-	-	23.62±8^T^^*^	21.91±8.01^T^
Female	21.80±7.97^T^	-	-	19.17±8.07^T^^*^	23.54±7.51^T^
Age (years)	-0.013^S^	-0.175^S^^*^	0.222^S^^*^	0.116^S^	-0.159^S^^*^
Weight (kg)	-0.342^S^^***^	-0.221^S^^*^	-0.481^S^^***^	-0.399^S^^**^	-0.313^S^^**^
Height (cm)	-0.393^P^^***^	-0.352^P^^**^	-0.443^P^^***^	-0.352^S^^**^	-0.451^S^^***^
BMI (kg/m2)	-0.168^S^^*^	-0.032^S^	-0.345^P^^**^	-0.280^P^^**^	-0.147^P^^*^
WC (cm)	-0.022^S^	0.152^S^	-0.205^S^^*^	-0.154^S^	0.052^S^
HC (cm)	-0.174^P^^**^	-0.142^P^	-0.208^P^^*^	-0.108^P^	-0.249^P^^**^
WC/HC	0.050^S^	0.033^S^	0.050^S^	0.078^S^	0.052^S^
WC/stature	0.190^S^^**^	0.283^S^^**^	0.088^S^	0.235^S^^*^	0.139^S^
Socio economic class					
A or B	21.74±7.85^T^	23.19±7.43^T^	20.25±8.13^T^^*^	20.23±7.89^T^	22.78±7.77^T^
C	22.60±8.08^T^	22.12±8.49^T^	23.13±7.71^T^^*^	22.55±8.57^T^	22.64±7.85^T^
Physical activity profile⊄					
Active	22.65±8.24^T^	22.86±8.45^T^	22.46±8.14^T^	21.46±8.31^T^	23.39±8.19^T^^*^
Sedentary	21.19±7.25^T^	22.13±7.23^T^	19.69±7.27^T^	21.51±8.43^T^	20.94±6.39^T^^*^
Quality of life					
Physical aspect	-0.314^S^^***^	-0.396^S^^***^	-0.208^S^^*^	-0.521^S^^***^	-0.168^S^^*^
Emotional aspect	-0.17^S^^*^	-0.108^S^	-0.137^S^	-0.144^S^	-0.079^S^
Social aspect	-0.072^S^	-0.139^S^	-0.009^S^	-0.132^S^	0.022^S^
Educational aspect	-0.053^S^	0.004^S^	-0.147^S^	-0.136^S^	0.032^S^

⊄ Data expressed as mean ± standard deviation of the AIx@75 in relation to the categories of the variable.

T with the Student's t-test for comparison of means. The other data are expressed as linear correlation coefficients (LCC).

P Pearson and

S Spearman (*=*p*<0.20, **=*p*<0.05 and ***=*p*<0.001. in the CCL significance test). BMI - body mass index; WC - waist circumference; HC - hip circumference.

**Table 4:** Linear correlation coefficient (LCC) between AIx@75 and measurements of peripheral and central arterial blood pressure. hemodynamic parameters and arterial stiffness.

		*Gender*		*Age group*	
Variables	Sample (n=134)	Male (n=69)	Female (n=65)	Child (n=53)	Adolescents (n=81)
Peripheral arterial pressure					
pSBP (mmHg)	-0.326^P^^***^	-0.270^P^^**^	-0.374^P^^**^	-0.416^P^^**^	-0.274^P^^**^
pDBP (mmHg)	-0.171^P^^*^	-0.067^P^	-0.316^P^^**^	-0.297^P^^**^	-0.084^P^
pMAP (mmHg)	-0.265^S^^**^	-0.168^S^^*^	-0.380^S^^**^	-0.411^S^^**^	-0.195^S^^*^
pPP (mmHg)	-0.211^P^^**^	-0.206^P^^*^	-0.205^P^^*^	-0.181^P^^*^	-0.264^P^^**^
Central blood pressure					
cSBP (mmHg)	-0.157^P^^*^	-0.173^P^^*^	-0.138^P^	-0.366^P^^**^	0.032^P^
cDBP (mmHg)	-0.167^P^^*^	-0.102^P^	-0.264^P^^**^	-0.282^P^^**^	-0.100^P^
Hemodynamics					
SV (mL)	-0.815^S^^***^	-0.831^S^^***^	-0.791^S^^***^	-0.837^S^^***^	-0.802^S^^***^
CO (L/min)	-0.549^S^^***^	-0.481^S^^***^	-0.602^S^^***^	-0.529^S^^***^	-0.578^S^^***^
TVR (s*mmHg/mL)	0.392^S^^***^	0.322^S^^**^	0.432^S^^***^	0.390^S^^**^	0.410^S^^***^
CI (L/min*L/m2)	0.160^S^^*^	0.152^S^	0.193^S^^*^	0.242^S^^*^	0.118^S^
Arterial stiffness					
PWV (m/s)	-0.253^S^^**^	-0.231^S^^*^	-0.268^S^^**^	-0.286^S^^**^	-0.240^S^^**^

SBP - Systolic blood pressure; DBP - Diastolic blood pressure; MAP - Mean blood pressure; PP - Pulse pressure; HR - Heart rate; PPA - Pulse pressure amplification; SV - Systolic volume; CO - Cardiac output; TVR - Total vascular resistance; CI - Cardiac index; PWV - Pulse wave velocity.

P Pearson and

S Spearman test

(*p<0.20, **p<0.05 and ***p<0.001 in the LCC significance test).

**Table 5:** Coefficients of the reference equations for the AIx@75.

Variables	Coefficient	CI 95%	*p*-value
All samples *(R^2^ = 80.47%)*			
Constant	44.017	(30.417; 57.617)	<0.001
Age	0.269	(0.072; 0.466)	0.008
pDBP	-0.225	(-0.346; -0.104)	<0.001
pMAP	0.134	(0.026; 0.242)	0.016
PPA	-14.478	(-18.176; -10.779)	<0.001
SV	-0.638	(-0.702; -0.573)	<0.001
CI	1.681	(0.469; 2.893)	0.007
PWV	6.936	(3.643; 10.228)	<0.001
Male sex *(R^2^ = 78.56%)*			
Constant	-9.952	(-37.778; 17.873)	0.477
pPP	0.292	(0.128; 0.456)	<0.001
SV	-0.621	(-0.727; -0.515)	<0.001
CI	2.847	(1.074; 4.620)	0.002
TVR	17.725	(7.827; 27.623)	<0.001
PWV	5.521	(0.683; 10.358)	0.026
Female sex *(R^2^ = 82.45%)*			
Constant	54.038	(38.156; 69.920)	<0.001
pDBP	-0.213	(-0.377; -0.049)	0.012
PPA	-15.652	(-20.087; -11.217)	<0.001
SV	-0.635	(-0.725; -0.545)	<0.001
PWV	9.376	(5.796; 12.956)	<0.001
Children (9 to12) *(R^2^ = 79.17%)*			
Constant	51.080	(27.791; 74.370)	<0.001
pDBP	-0.274	(-0.449; -0.099)	0.003
PPA	-11.936	(-19.112; -4.759)	0.002
SV	-0.662	(-0.770; -0.553)	<0.001
PWV	9.931	(4.754; 15.109)	<0.001
Adolescents (13 a 19 anos) *(R^2^ = 81.57%)*			
Constant	35.683	(16.393; 54.973)	<0.001
BMI	-0.369	(-0.697; -0.041)	0.028
pDBP	-0.313	(-0.496; -0.130)	0.001
PPA	-11.591	(-15.978; -7.203)	<0.001
SV	-0.590	(-0.673; -0.507)	<0.001
TVR	15.294	(6.373; 24.215)	0.001
PWV	10.732	(6.778; 14.686)	<0.001

pDBP - Peripheral diastolic blood pressure; pMAP - Peripheral mean blood pressure; PPA - Pulse pressure amplification; SV - Systolic volume; CI - Cardiac index; PWV - Pulse wave velocity; pPP - Peripheral pulse pressure; TVR - Total vascular resistance; BMI - Body mass index.
